# Predictability effects in auditory scene analysis: a review

**DOI:** 10.3389/fnins.2014.00060

**Published:** 2014-03-31

**Authors:** Alexandra Bendixen

**Affiliations:** Auditory Psychophysiology Lab, Department of Psychology, Cluster of Excellence “Hearing4all,” European Medical School, Carl von Ossietzky University of OldenburgOldenburg, Germany

**Keywords:** predictive coding, sound processing, auditory stream segregation, integration, bistable perception, old-plus-new heuristic

## Abstract

Many sound sources emit signals in a predictable manner. The idea that predictability can be exploited to support the segregation of one source's signal emissions from the overlapping signals of other sources has been expressed for a long time. Yet experimental evidence for a strong role of predictability within auditory scene analysis (ASA) has been scarce. Recently, there has been an upsurge in experimental and theoretical work on this topic resulting from fundamental changes in our perspective on how the brain extracts predictability from series of sensory events. Based on effortless predictive processing in the auditory system, it becomes more plausible that predictability would be available as a cue for sound source decomposition. In the present contribution, empirical evidence for such a role of predictability in ASA will be reviewed. It will be shown that predictability affects ASA both when it is present in the sound source of interest (perceptual foreground) and when it is present in other sound sources that the listener wishes to ignore (perceptual background). First evidence pointing toward age-related impairments in the latter capacity will be addressed. Moreover, it will be illustrated how effects of predictability can be shown by means of objective listening tests as well as by subjective report procedures, with the latter approach typically exploiting the multi-stable nature of auditory perception. Critical aspects of study design will be delineated to ensure that predictability effects can be unambiguously interpreted. Possible mechanisms for a functional role of predictability within ASA will be discussed, and an analogy with the old-plus-new heuristic for grouping simultaneous acoustic signals will be suggested.

## Introduction: auditory scene analysis principles

Our auditory system is often confronted with a mixture of sounds originating from several different sources. Relevant auditory information can only be retrieved if the system succeeds in decomposing this mixture into meaningful perceptual units termed *streams* (Bregman, 1990). Originally introduced as the *cocktail party problem* (Cherry, 1953), the sound stream decomposition problem was later coined *auditory scene analysis (ASA)* in an influential monograph by Bregman (1990). Theories and computational models of ASA increasingly incorporate structural and functional knowledge about the auditory system coming from other research areas (e.g., Haykin and Chen, 2005). In this spirit, the present contribution will link *predictive processing*, a central property of the auditory system extensively investigated in the neurosciences (e.g., Friston, 2005, 2010), with an important principle of ASA, the *old-plus-new heuristic*. On theoretical and empirical grounds, it will be argued that the formation and maintenance of stable sound source representations is facilitated by the capacity of the auditory system to extract predictability from the sound sources' signal emissions.

Disentangling a sound mixture requires inferring likely sources from physically overlapping signals. Two different types of signal decomposition are needed. First, for signals occurring at the same time, the listener must interpret whether the same or different sound source(s) emitted them. This process, called *concurrent* or *vertical grouping* (Bregman, 1990), rests on auditory cues such as location, harmonicity, and onset synchrony of the different components of a mixture (e.g., McDonald and Alain, 2005; Lipp et al., 2010; for review, see Micheyl and Oxenham, 2010b). As these cues are immediately informative of the possible relations between co-occurring auditory signals, they are called *simultaneous* or *instantaneous* cues.

Other cues carry no information in and of themselves, but are informative only in comparison with previous input. One such cue might be that several tones in succession all occupy the same frequency region. This second type of signal decomposition is termed *horizontal* or *sequential grouping* (Bregman, 1990). It is concerned with interpreting the relations between different auditory signals following each other in time. Again, the listener must interpret whether these signals were emitted by the same or different sound source(s). The focus of the present contribution is on this second situation, where the ambiguity lies in the spreading out of sound signals over time rather than in their physical overlap at one particular moment.

Indeed, many natural sound sources emit signals in a temporally discontinuous manner (e.g., a series of footsteps, or an utterance containing speech-inherent pauses). The auditory system of the listener is then confronted with discrete sound events that need to be bound together (*stream integration*). At the same time, binding events together that were actually emitted by two different sources needs to be avoided (*stream segregation*). The perceptual decision as to whether sounds in a mixture should be grouped together or not has been suggested to be based on a number of heuristics, many of them originating from the Gestalt school of psychology (e.g., Wertheimer, 1923; Köhler, 1947). The feature *similarity* principle posits that sounds are more likely to have been emitted by the same source if they are similar in all of their acoustic features (for detailed discussion, see Moore and Gockel, 2002, 2012). Feature (dis)similarity is further evaluated against the temporal separation between consecutive sound events (Van Noorden, 1975) as sound sources rarely change the features of their sound emissions abruptly, but may do so over time. This relation can be expressed in quantitative terms by the temporal rate of feature change (Jones, 1976). Feature similarity can then be quantified as the inverse of this rate (Winkler et al., 2012; Mill et al., 2013). In doing so, the Gestalt rule of *similarity* merges into the principle of *continuity*, expressing the notion that sound sources show smooth variation over time rather than abrupt changes (Bregman, 1990).

Another important principle of ASA is called the *old-plus-new heuristic*. This heuristic was formulated by Bregman (1990, p. 222; going back to Helmholtz, 1859, p. 59f.) to express the idea that the auditory system, when confronted with a mixture of sounds, first looks for continuations of previous sounds and removes these from the mixture, and then analyzes the residue. According to Bregman's (1990) description, “mixture” in this case refers to the actual physical overlap of sounds; and “continuation” means uninterrupted continuation, i.e., one and the same sound that goes on while other sounds are added. With these specifications, it becomes evident that the old-plus-new heuristic is a cue for vertical (simultaneous) sound grouping. One may, however, easily have the idea that the same could be true for interrupted (i.e., discrete) forms of continuation, such as a sound source emitting the same sound event regularly every so many milliseconds (ms). An illustrative example is the repetitive acoustic signature of a train moving on the rails. In this situation, giving the continuation of old sound sources (the train sound) precedence over the identification of new sources (e.g., someone opening the cabin door) would again lead to a plausible decomposition of the auditory scene into known (old) and unknown (new and potentially relevant) information. The old-plus-new heuristic would then transfer from vertical to horizontal (sequential) sound grouping.

The Gestalt principles of *similarity* and *continuity* appear conceptually similar to the old-plus-new heuristic, in that they express the idea that a sound source continues with relatively unchanged attributes. However, in fact they do not constitute a sequential analogue to the old-plus-new heuristic. Instead, they are “unspecific” variants of the old-plus-new heuristic in that they do not distinguish between exact continuation and inexact but plausible continuation of the sound source's behavior (Bregman, 1990). In contrast, the old-plus-new heuristic appears to be based on the system assuming a very precise continuation for uninterrupted sounds (Darwin, 1995). Furthermore, the old-plus-new heuristic implies that sound continuations are subtracted from a mixture before running any other decomposition algorithms. In contrast, continuity does not take precedence over other grouping cues: The plausibility of continuation is checked only *after* the sequence has been partitioned (Rogers and Bregman, 1993). Hence according to Bregman's (1990) framework, vertical sound grouping appears to be equipped with a more powerful mechanism of dealing with signal continuation than horizontal sound grouping. This might be partly due to the fact that at the time, much less was known about the auditory system's capacity to detect continuity in a sequence of discrete sounds. Within the last two decades, this topic has experienced an upsurge in interest and research activity, leading to a much more comprehensive picture of how continuous (regular) properties can be extracted from discrete sounds.

## Predictive auditory processing

The concepts and empirical findings outlined in this section initially developed independently from ASA research. They were based on the experimental observation (Näätänen et al., 1978) and later theoretical conceptualization (Näätänen, 1992) of the auditory system's capacity to detect deviations from otherwise constant features in a sequence of discrete sounds. In a typical study, single tones of constant frequency (*standards*) would be repetitively presented. Occasionally, the frequency of one tone would be changed. These *deviant* tones would elicit a specific brain response originating in auditory cortical areas (Giard et al., 1990; Opitz et al., 2002): the mismatch negativity (MMN) component of the event-related potential (ERP). MMN was interpreted to indicate “mismatch” or *deviance* detection in otherwise regular sound sequences (Näätänen et al., 1978; see also Snyder and Hillyard, 1976).

Since the detection of deviant sounds requires the prior recognition of an invariant property in the standard sounds (e.g., their constant frequency), the argument went on to suggest that MMN elicitation can be taken as indirect evidence of *invariance* extraction (e.g., Picton et al., 2000). Following observations that not only constant feature values but also feature values changing in a regular manner are encoded as standards (e.g., alternation between feature values, “ABAB…”), the term “invariance” was replaced by “*regularity*” (Winkler, 2007). This leads to the notion of “regularity extraction” from sound sequences, which is equivalent to the detection of continuous properties in a source's discrete signal emissions referred to above. The types of regularities that can be extracted have been intensely studied, demonstrating the enormous potential of the auditory system to detect relations of various degrees of complexity between successive sounds in a sequence (cf. reviews by Näätänen et al., 2001, 2010).

Finally, the notion was put forward that regularity extraction is conceptually identical to the extraction of *predictability* from a sound sequence (Tiitinen et al., 1994; Winkler et al., 1996). It was further argued that the extracted information is used for *predicting* upcoming sounds (Baldeweg, 2006). This notion has gained momentum with the advent of predictive coding theory (Friston, 2005, 2010). Processing sensory input in a predictive manner has become an important element of general theories of perception (Gregory, 1980; Friston, 2005, 2010; Prinz, 2006; Schubotz, 2007). Abundant empirical evidence for predictive processing in the auditory system has been gathered, which is partly based on MMN and partly on more direct ERP indicators (for a recent review, see Bendixen et al., 2012a).

Whereas there is not yet consensus as to the precise underlying neuronal mechanisms and the terminology best used to describe the relevant phenomena (e.g., Näätänen et al., 2005; May and Tiitinen, 2010), it is undisputed that the auditory system effortlessly acquires information about the regular structure of the surrounding sound sources. The term “effortlessly” is meant to imply that this information comes as an inherent property of auditory sensory information processing (as opposed to being made available only by actively searching for it). There is also general agreement that information about the regular characteristics of sound sources is available to the auditory system at early processing stages—within 150 ms after sound onset, when considering the latency of the MMN. More recent findings imply that the information is available even earlier, within 20–40 ms after sound onset (Grimm et al., 2011; cf. review by Escera et al., in press). In fact, the predictive notion implies that such information should be available even *before* the onset of the next signal emitted by a given sound source (Baldeweg, 2006; Bendixen et al., 2009, 2012a).

## Implications of predictive processing for auditory scene analysis

The early availability of information on the regular characteristics of sound sources implies that predictability could, in theory, be used as an early cue in ASA. If auditory input is indeed processed in a predictive manner, information about the predictable succession of sound events could act upon ASA processes before any other grouping cue would be able to exert its influence. This is because all other cues need at least some rudimentary analysis of stimulus input, whereas prediction-based grouping could start at or even before the expected time of stimulus arrival. Therefore, predictability could in theory be the earliest grouping cue in ASA. Predictability would then constitute a sequential analogue to the old-plus-new heuristic in simultaneous sound grouping.

Such considerations have led some researchers to propose theories linking predictive processing and sequential ASA (Denham and Winkler, 2006; Winkler, 2007; Winkler et al., 2009). It should be pointed out that in previous theoretical frameworks, the impact of predictive regularities on ASA was not negated altogether but was ascribed to high-level, “schema-based” ASA processes requiring the involvement of attention (Bregman, 1990, p. 411ff.). The idea of an attention-mediated role of predictability within ASA was strongly driven by the work of Mari Riess Jones and her colleagues on rhythmic attending (Jones, 1976; Jones et al., 1981, 1982; Jones and Boltz, 1989; Drake et al., 2000). It was, for instance, shown that an immediately apparent regularity (e.g., rhythmicity) in the emissions of a sound source helps listeners to focus their attention on that sound source. The impact of the regularity was, however, regarded as confined to a second stage of ASA, where top-down selections between the groupings offered by the first stage are made (Bregman, 1990; Bey and McAdams, 2002). In contrast, the possibility of predictability entering low-level, “primitive” ASA processes was distinctly ruled out (Bregman, 1990, p. 135f.; cf. p. 417ff. for a summary of the arguments). It seems justified to re-examine this conclusion based on the insights into auditory predictability extraction that have been gained within the last 20 years.

One might argue that the insights into predictive processing are not transferrable to ASA because almost all findings were obtained in artificially simplified experimental situations with only one active sound source. Indeed, the vast majority of MMN studies employed only one sequence of sounds in which a single regularity was embedded. Yet some studies have shown that the MMN-eliciting system can monitor regularities in at least three sound streams simultaneously (Nager et al., 2003; Winkler et al., 2003c; Sussman et al., 2005). Together with the complex forms of predictability that can be discovered without attentional involvement (Näätänen et al., 2001, 2010), it seems that supposedly “primitive” auditory analyses can process much more complex scenarios than previously assumed (Nelken, 2004).

Consequently, the hypothesis has been put forward that predictability contributes to the initial decomposition of the auditory input, and that the predictive models underlying MMN generation form the basis of the sequential grouping processes underlying ASA (Denham and Winkler, 2006; Winkler, 2007; Winkler et al., 2009). This idea nicely converges with the fact that predictive elements have successfully been implemented in computational modeling approaches of ASA (Ellis, 1999; Godsmark and Brown, 1999; Masuda-Katsuse and Kawahara, 1999; Grossberg et al., 2004; Coy and Barker, 2007). One must, however, concede that a functional role of predictability within ASA has not received much empirical support. Indeed, the results of early ASA studies (French-St. George and Bregman, 1989; Rogers and Bregman, 1993) suggest quite the opposite: that sound predictability does *not* affect auditory stream segregation or integration. These early studies will be examined in the following.

## Early studies on predictability effects in auditory scene analysis

**Figure 1:**
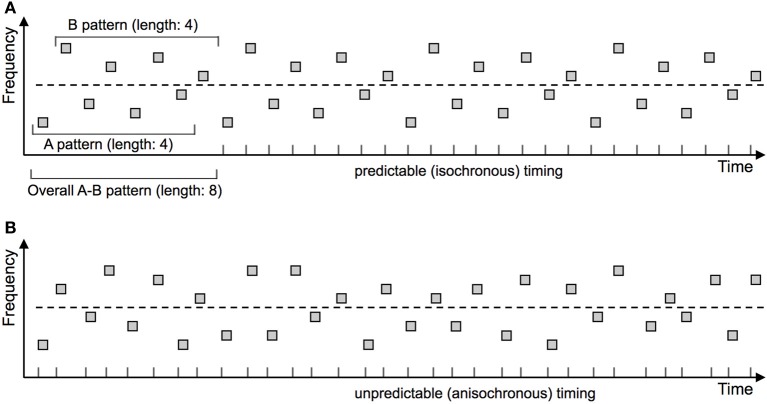
**Experimental paradigm confounding predictability of the “Integrated” and “Segregated” perceptual organizations**. French-St. George and Bregman (1989) compared stimulus arrangements that were predictable or unpredictable with respect to stimulus timing and frequency. Sequences were predictable on both dimensions **(A)**, on neither dimension **(B)**, or on just one of the dimensions (not depicted). The cyclically repeating (thereby predictable) frequency patterns are marked in the upper panel. Stimulus timing is additionally marked by corresponding ticks on the X axis. The dashed line indicates the (nominal) separation between the “A” and “B” groups of tones. Participants were asked to try perceiving all tones as originating from one sound source and to press a button as long as they succeed in maintaining this percept. Predictability was assumed to increase the perceptual coherence of the tone set, thereby leading to a higher probability of perceiving the sequence as one stream (“Integrated”). Yet as illustrated in the upper panel, adding predictability to the sequence as a whole unavoidably renders the two separate streams more predictable, too. Thus their individual perceptual coherence might increase as well, leading to a higher probability of perceiving the sequence as “Segregated.” These opposite effects might cancel each other out, leading to a null effect on average that would not be indicative of a general absence of predictability effects in ASA.

An influential study taken to demonstrate that ASA is unaffected by predictability was carried out by French-St. George and Bregman (1989). These authors presented a sequence of regularly alternating “A” and “B” sounds (i.e., “ABABABAB”), where “A” and “B” denote categories of sounds that could take one of four different frequency values each (A1–A4, B1–B4). The order of sounds was either arranged in a predictable manner (e.g., continuous repetition of the eight-tone cycle “A1B4A3B2A2B3A4B1”) or chosen randomly anew for each eight-tone cycle (see Figure 1 for illustration). Moreover, the delivery of the sounds was either isochronous (thus temporally predictable), or non-isochronous with unpredictable temporal intervals. The authors investigated whether these two manipulations of sound predictability would have an impact on participants' ability to perceive all the sounds together in one stream (“Integrated”) as determined by subjective perceptual report. Results revealed that neither of the predictability manipulations had any effect on the perception of the sequence (French-St. George and Bregman, 1989). The authors concluded that predictability does not support the perceptual coherence of a putative sound stream.

These results were taken to imply that ASA is unaffected by sound predictability (French-St. George and Bregman, 1989). There is, however, a crucial confound in the experimental design as noted by the authors themselves (p. 386): Although the predictability manipulation was designed to pertain to the whole sequence of sounds (“ABABABAB”), this co-varied with the predictability of the two sub-streams (“A-A-A-A-” and “-B-B-B-B” separately). Therefore, if predictability acts as a cue favoring decompositions with predictable behavior of the resulting sound sources, in this specific design predictability would have favored not only the “Integrated” but also the “Segregated” perceptual organization. The null effect observed by the authors might have been due to these opposite effects canceling each other out. The authors suggest that experiments with independent manipulations of the predictability of the overall sequence and of the separate sub-streams are needed (French-St. George and Bregman, 1989).

A later study by Rogers and Bregman (1993) manipulated predictability in a different way, by presenting an induction sequence before the test sequence to be perceptually judged by the listener (“A_A_A_A_A_ABA_ABA_ABA,” where “A_A_A” is the induction sequence, and “ABA_” is the test sequence). Predictability of the induction sequence was manipulated in terms of the occurrence times and duration values of the “A” stimuli. Consistent with the results of French-St. George and Bregman (1989), this manipulation had no effect on perceptual judgments of the test sequence (Rogers and Bregman, 1993). Yet again, a predictable induction sequence rendered not only the “A” tones of the test sequence, but also the whole “ABA_” pattern of the test sequence more predictable. Therefore, predictability may have supported both the “Integrated” and “Segregated” perceptual organizations, and the two effects may have canceled each other out. Consequently, empirical evidence in favor of or against sound predictability effects in ASA is inconclusive based on these early studies.

## De-confounding predictability effects on stream segregation and integration

Some further studies investigating the role of predictability within ASA emerged recently, motivated by the increased interest in auditory predictive processing (Bendixen et al., 2010, 2012b, 2013a, in revision; Devergie et al., 2010; Andreou et al., 2011; Rimmele et al., 2012; Rajendran et al., 2013). In these studies, care was taken not to introduce a confounding effect of influencing stream segregation and integration in parallel. To avoid this confound, one needs to acknowledge that it is difficult to manipulate a tone sequence such that only one of the perceptual organizations (but not the other) would change in terms of *formal* (mathematical) predictability. Yet it is much easier to achieve such a manipulation when considering *perceptual* rather than formal predictability. In some cases, formally predictable tone sequences are treated as if they were unpredictable by the auditory system because the predictable pattern contains too many elements or spans too much time, thereby exceeding memory limitations (e.g., Scherg et al., 1989; Sussman et al., 1999; Sussman and Gumenyuk, 2005; Boh et al., 2011). This knowledge can be exploited for introducing manipulations that render only the “Integrated” or only the “Segregated” perceptual organization predictable.

**Figure 2:**
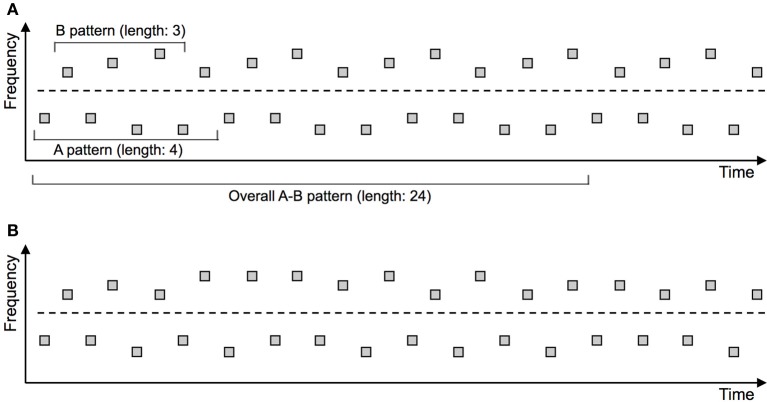
**Experimental paradigm disentangling predictability of the “Integrated” and “Segregated” perceptual organizations**. The depicted stimulus arrangements are predictable **(A)** or unpredictable **(B)** with respect to stimulus frequency. The cyclically repeating (thereby predictable) frequency patterns are marked in the upper panel. The dashed line indicates the (nominal) separation between the “A” and “B” groups of tones. Critically, the number of elements included in the predictable patterns differs between the “A” and “B” group of tones. As a result, the length of the cyclically repeating overall pattern comprising “A” and “B” tones amounts to 24 elements, which is considerably too long to be picked up by the auditory system (e.g., Boh et al., 2011). Consequently, from a perceptual point of view the predictability manipulation is *directional*: It affects only predictability of the “Segregated” perceptual organization, whereas predictability of the “Integrated” organization remains unchanged. This directional manipulation allows for an unambiguous investigation of predictability effects in ASA (e.g., Bendixen et al., 2010, 2013a).

An example of such an independent manipulation is depicted in Figure 2. The predictability manipulation is based on changing the sequential linkage between successive tones within each set (“A-A-A” and “B-B-B”) or across the sets (“A-B” and “B-A”), such that these sounds are predictive of each other in some conditions but not in other conditions. Sequential links within each set affect the predictability of the “Segregated” organization, while links across sets affect the predictability of the “Integrated” organization. Achieving one without the other leads to a *directional* predictability manipulation, avoiding the above-mentioned confound.

As depicted on Figure 2, one can include a regular pattern of a particular length (e.g., 4 elements) into the “A” sub-sequence, and a separate pattern of a different length (e.g., 3 elements) into the “B” sub-sequence (e.g., “B1B2B3B1B2B3…,” with 1/2/3 representing slightly different frequency values within the “B” stream). Compared to a random arrangement of frequencies (e.g., “B1B1B3B1B2B3B2…”), this enhances the predictability of the “B” sub-sequence (and, by the same principle but independently, of the “A” sub-sequence). Hence the predictability of the “Segregated” perceptual organization increases. From a formal-mathematical point of view, predictability of the whole “AB” sequence (and hence of the “Integrated” organization) also increases, but the resulting pattern spans 24 elements, which is considerably too long to be picked up by the auditory system (e.g., Boh et al., 2011). Therefore, from a perceptual point of view, predictability of the “Integrated” perceptual organization remains unchanged. This type of manipulation thus selectively increases predictability of the “Segregated” perceptual organization, permitting a clear inference as to the role of predictability within ASA (Bendixen et al., 2010, 2013a).

Selectively manipulating predictability of the “Segregated” perceptual organization can also be achieved by changing one of the sub-sequences (only “A” or only “B”) from random to predictable, while leaving the other sub-sequence random (Devergie et al., 2010; Andreou et al., 2011; Rimmele et al., 2012). In this case, predictability of the “Integrated” organization again remains unchanged, while the “Segregated” organization becomes partially predictable.

A closely related possibility is to start with two predictable sub-sequences and to then change one of the sub-sequences (only “A” or only “B”) from predictable to random, while leaving the other sub-sequence predictable (Bendixen et al., 2012b; Rajendran et al., 2013). Provided that the regular patterns of the two predictable sub-sequences had been distinct from each other to preclude predictability of the whole sequence (“Integrated” organization), this would again lead to a selective manipulation of predictability of the “Segregated” organization (Bendixen et al., 2012b). If, on the other hand, the regular patterns of the two predictable sub-sequences had rendered the whole sequence predictable (e.g., because they were identical in length, forming an easily detectable overall pattern), changing one of the sub-sequences would decrease predictability of the “Integrated” and “Segregated” organizations in parallel, making the results of the predictability manipulation more difficult to interpret (Rajendran et al., 2013). This problem pertains to any design where the “predictable” condition is chosen to have no feature variation in the “A” and “B” sequences (as in the classical “ABA_” paradigm; Van Noorden, 1975). Constancy is of course the easiest form of predictability, but it is also the one that is most difficult for disentangling “Segregated” and “Integrated” predictability.

In summary, by exploiting knowledge on the pattern lengths that the auditory system recognizes as predictable, it is feasible to selectively manipulate predictability of the “Segregated” perceptual organization. The opposite case, manipulating predictability of the “Integrated” but not “Segregated” perceptual organization, is more difficult to achieve. Consider that the whole “ABA_” sequence, corresponding to the “Integrated” organization, is designed to be predictable. In this case, the feature values of the first “A” tone predict those of the “B” tone, while the feature values of “B” in turn predict those of the second “A” tone. In order to avoid predictability of the “Segregated” organization, one would have to ensure that the feature values of the first “A” tone are not predictive of those of the second “A” tone. This is difficult, if not impossible to achieve when the predictability manipulation is based on only one feature. In a recent study, we proposed a compromise solution (Bendixen et al., in revision) where the “Integrated” organization is at least partially predictable, while the “Segregated” organization remains almost entirely unpredictable.

**Figure 3:**
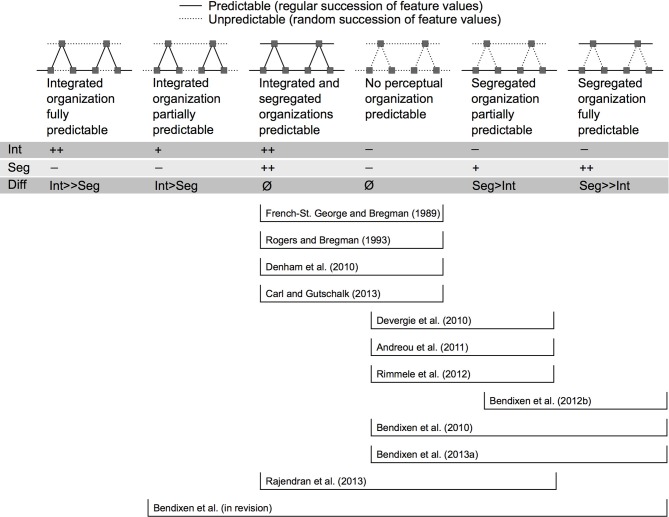
**Designs for studying predictability effects in auditory scene analysis**. Six different levels of predictability are distinguished; and it is indicated for each of the previous studies which levels they have contrasted. Each level is schematically illustrated with a cutout of the corresponding stimulus sequence. Time is represented on the X axis and frequency on the Y axis of all panels. The schematic depiction uses the “ABA_” paradigm (Van Noorden, 1975), but studies have also used the “ABAB” paradigm or more irregular arrangements of “A” and “B” tones. Straight lines indicate the presence of predictive relations between successive tones (i.e., the feature values of one tone are predictive of the feature values of the next tone in one or both perceptual organizations). Dotted lines indicate random successions of feature values. The feature whose predictability was manipulated differs between studies (e.g., onset time, frequency, intensity, location). The effects on predictability of the “Integrated” (Int) and “Segregated” (Seg) organizations are marked with “++” (fully predictable), “+” (partially predictable), or “−” (unpredictable). The resulting predictability difference between the two organizations is marked in the “Diff” row. Following these differences, predictability conditions to the left should increase the likelihood of “Integrated” percepts, whereas predictability conditions to the right should increase the likelihood of “Segregated” percepts. Note that many studies have compared conditions in the middle of this scheme, and have revealed no clear effects of predictability on auditory perceptual organization. Studies employing directional manipulations have tended to investigate conditions where the “Segregated” organization was more predictable than the “Integrated” organization (depicted on the right of this scheme). No study has so far investigated a condition in which the “Integrated” but not the “Segregated” organization was fully predictable.

The various possibilities of manipulating predictability of the “Segregated” or “Integrated” organization (sometimes unwantedly affecting both in parallel) are summarized in Figure 3, alongside with studies that exemplify the resulting comparisons. Six different predictability conditions are distinguished on Figure 3. In the two conditions to the left, predictability of the “Integrated” organization is higher than predictability of the “Segregated” organization. The converse is true for the two conditions to the right. The two conditions in the middle represent cases where predictability of the “Integrated” and “Segregated” organizations do not differ (they are either both perfectly predictable or both unpredictable). As a consequence, empirical investigations comparing these two middle conditions must be regarded as uninformative with respect to the role of predictability as a cue in ASA. The fact that such studies (French-St. George and Bregman, 1989; Rogers and Bregman, 1993; Denham et al., 2010; Carl and Gutschalk, 2013) revealed no clear effects of the predictability manipulation can be attributed to the above-mentioned confound. It should be noted, though, that some of these studies employed the predictability manipulation with a different aim than the one in focus here, and that their results are nevertheless informative for models of ASA in a wider sense (see below).

Results of the studies changing predictability in a directional manner will be examined in the following. If the proposition that predictability contributes to the organization of the auditory scene is correct (Denham and Winkler, 2006; Winkler, 2007; Winkler et al., 2009, 2012), the empirical results should show an increase in the likelihood of stream segregation for those conditions where the predictability of the “Segregated” organization is selectively increased. In turn, the likelihood of stream integration should increase for those conditions where the predictability of the “Integrated” organization is selectively increased. Before reviewing the empirical data, the methods typically employed for studying ASA will be briefly summarized to point at some methodological advances that have contributed to the findings under review.

## Methodological aspects of measuring auditory scene analysis

Two main approaches are typically pursued to study ASA (Bregman, 1990; Micheyl and Oxenham, 2010a; Spielmann et al., 2013). Participants can be asked explicitly how they perceived a given auditory scene; in particular, whether they heard one or two sound sources at each particular moment (*subjective-report procedure*). This procedure has the advantage of providing a direct measurement of perceptual organization. However, it comes with the disadvantage of any introspective method: the limited possibility to validate whether participants are indicating their perception in a veridical manner. It is therefore beneficial to complement the subjective-report data with a second approach (*objective-listening tests*): Listening tasks can be set up that are easier to accomplish if participants are perceiving the auditory scene in one way (e.g., segregated into two sound sources) rather than another (e.g., integrated into one sound source). Poor task performance is then indicative of the listener not being able to segregate (or, in tasks applying the opposite logic, to integrate) the streams despite trying to do so volitionally. These tests have the advantage of permitting objective measurements, yet the disadvantage of being but indirect measures of the actual percept. It is, for instance, possible that participants succeed in stream segregation but still fail to perform the task on the foreground stream because they are too heavily distracted by the presence of the background stream. Perhaps more often neglected, the opposite is also possible: Participants might succeed in performing the task although they do not succeed in segregating the streams. This happens if participants find a way of solving the task without perceptually organizing the auditory scene in the way the experiment was set up to evoke. For instance, it has been reported that participants can adopt the strategy of listening out for every other tone in a mixture to circumvent the need of stream segregation (Dowling et al., 1987). In both cases, task performance would become an invalid indicator of perceptual organization. The two approaches offered by objective-listening tests and subjective-report procedures can therefore be seen as complementary in the type of evidence they provide as well as in the type of alternative explanations they can rule out.

Recent advances regarding the objective-listening tests have been brought about by improved analysis methods for assessing task performance under fast presentation conditions (Bendixen and Andersen, 2013). Moreover, it was demonstrated that objective-listening performance (and hence, success in stream segregation or integration) can be measured not only behaviorally but also in terms of ERPs, with close correspondence between these measures (Sussman et al., 2001; Winkler et al., 2003b). This reduces the reliance on giving participants a behavioral task, and thereby considerably widens the scope of studying ASA (e.g., toward ASA processes outside the focus of attention, cf. Sussman et al., 2007; or toward participant groups with difficulties in giving behavioral responses, cf. Winkler et al., 2003a). It also permits parallel acquisition of objective indicators of stream segregation and integration (Spielmann et al., in press) to be able to cross-validate these indirect measures of perception.

New methodological insights have also been gained throughout the past years in terms of the subjective-report procedure. It has been demonstrated that the perception of one vs. two streams is not fully determined by the temporal and feature characteristics of the stimulus sequence. Instead, stream perception underlies inter-individual and, more strikingly, intra-individual fluctuations. When presenting cyclically repeating “ABA_” sequences and asking participants to report their current percept in a continuous manner, perception switches back and forth between the “Integrated” and “Segregated” alternatives (and possibly more, cf. Denham et al., 2014). Although initial attempts with this method (Anstis and Saida, 1985) have reported that perception settles on the “Segregated” interpretation rather than switching between the alternatives, more recent findings converge in showing that stochastic perceptual switching does occur with prolonged exposure times (e.g., Gutschalk et al., 2005; Winkler et al., 2005; Kondo and Kashino, 2009; Hill et al., 2011). Such changes in perception despite unchanged stimulus configuration constitute a case of bi- or multi-stability in audition that is analogous to bi-stable phenomena in vision (Pressnitzer and Hupé, 2006; Hupé and Pressnitzer, 2012; Kondo et al., 2012). Fluctuations between alternative percepts occur for a wide range of the acoustical parameters (Denham et al., 2013). They probably reflect the fact that the auditory system explores different alternatives of grouping the sounds (Denham and Winkler, 2006; Winkler et al., 2009, 2012; Denham et al., 2014).

**Figure 4:**
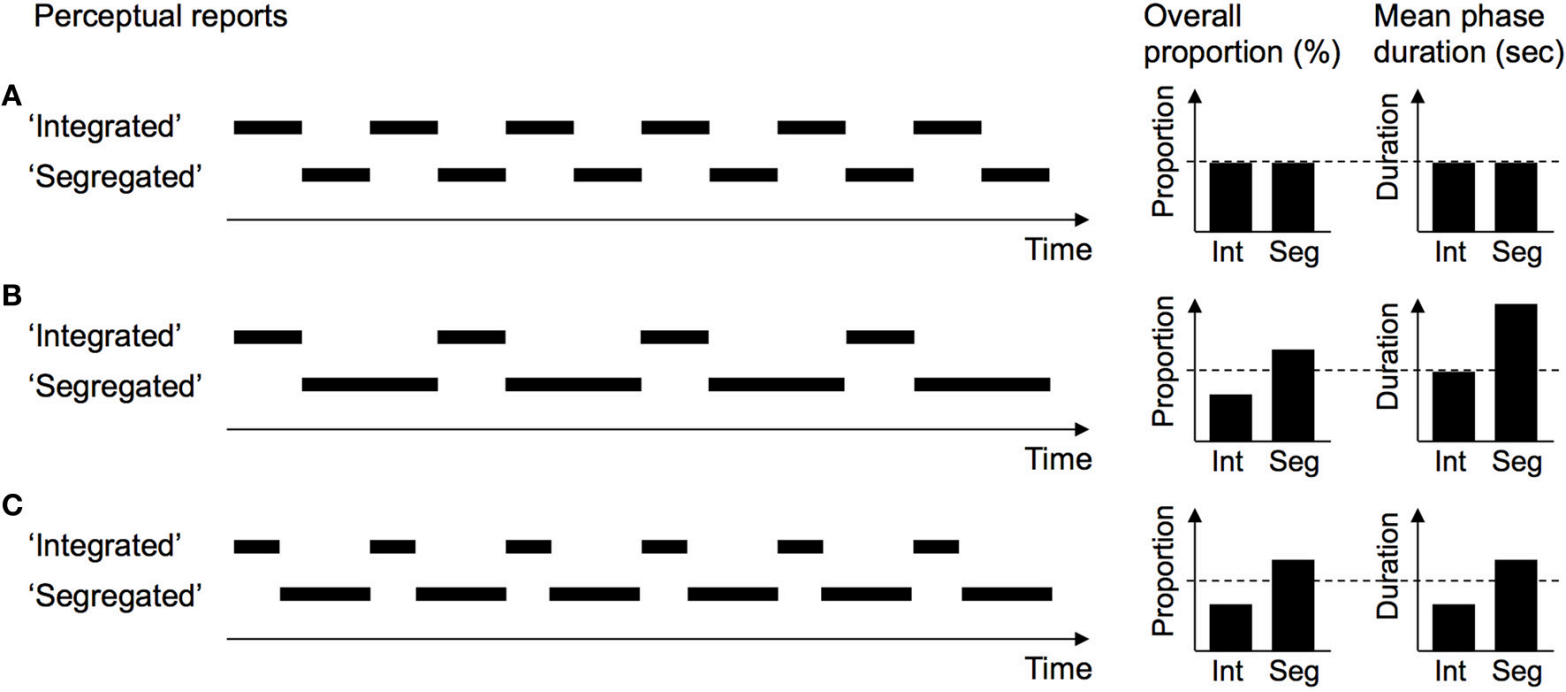
**Possible cue effects in the subjective-report procedure**. Artificially simplified time-courses of perceptual switching were generated for illustration purposes. The upper row **(A)** reflects a balanced distribution of “Integrated” and “Segregated” percepts. The middle row **(B)** shows the impact of a percept-stabilizing cue in favor of stream segregation, which prolongs the duration (i.e., stability) of “Segregated” percepts but leaves the duration of “Integrated” percepts unaffected. The lower row **(C)** shows the impact of a percept-inducing cue in favor of segregation, which prolongs the duration of “Segregated” percepts and additionally shortens the duration of “Integrated” percepts by causing perceptual switches back to the “Segregated” percept. The dashed lines in each panel mark the proportion and duration values from the balanced condition for comparison. Note that in this example, percept-stabilizing and percept-inducing cues have identical effects on the proportions of the two percepts, hence analyzing only the average proportions cannot differentiate between these qualitatively different types of cues. The average phase durations are informative about the cause of the changes in proportion, and thus about the underlying mechanism of the cue.

The bi-stable nature of perception in the auditory streaming paradigm is becoming a popular tool in ASA research (cf. Pressnitzer et al., 2011). It can, for instance, be exploited for a characterization of the impact of different cues affecting ASA. By analyzing the time-course of perceptual switching, it is possible to separate cues that initiate perceptual switches from cues that stabilize a given percept but do not cause switching toward it (e.g., Bendixen et al., 2010, 2013a,b; Szalárdy et al., 2013, in press). The latter cues prolong the duration of experiencing a given percept, while the former cues additionally shorten the duration of experiencing the alternative percept(s). Both types of cue effects lead to an increase in the overall proportion of experiencing the associated percept (see Figure 4 for illustration). The differentiation of the underlying mechanism speaks to the stage of ASA at which the cues exert their influence. Cues that can be shown to initiate switches must exert their influence at an early (partitioning) stage of ASA, at which the decomposition is initially decided upon. In contrast, for cues that merely stabilize a given percept but cannot initiate switches toward it, it can be inferred that they are not able to affect this early stage but are limited to providing feedback to a perceptual organization emerging spontaneously or by means of other cues.

## Recent studies on predictability effects in auditory scene analysis

With these new methodological prospects, the issue of predictability as a cue in ASA was revisited by some recent studies. In the majority of studies (Bendixen et al., 2010, 2013a; Devergie et al., 2010; Andreou et al., 2011; Rimmele et al., 2012), predictability of the “Segregated” organization was selectively manipulated. A selective increase in “Segregated” predictability was tested as an additional cue toward stream segregation, while a primary cue for stream segregation (in particular, spectral separation) was also present. The precise manipulations as well as the employed control conditions differed between studies, but when sorting them into the scheme developed in Figure 3, it becomes obvious that all of them contrasted one condition where the “Segregated” organization was more predictable than the “Integrated” organization with another condition where the two organizations were equally (un)predictable (note that this is also true for the study of Rajendran et al., 2013, though interpreted in a different framework by the authors). Notably, all the aforementioned studies found a higher proportion of stream segregation in those conditions where the predictability of the “Segregated” organization was selectively enhanced. These consistent results strongly support the view that predictability is used as a cue in ASA.

The result that the predictability of the “Segregated” organization enhances stream segregation was obtained both with subjective-report procedures (Bendixen et al., 2010, 2013a; Rajendran et al., 2013) and with objective-listening tasks (Devergie et al., 2010; Andreou et al., 2011; Rimmele et al., 2012). Hence predictability effects occur with neutral listening instructions (i.e., they bias perception of ambiguous auditory scenes toward configurations with higher predictability) as well as with active attempts to segregate the streams for solving a given task. Moreover, in the objective-listening tasks, predictability supported stream segregation both when it was embedded in the stream that the listener was instructed to focus upon (Rimmele et al., 2012; cf. earlier work by Jones et al., 1981) and when it was embedded in another stream that was to be ignored by the listener (Devergie et al., 2010; Andreou et al., 2011; Rimmele et al., 2012). In other words, predictability of one sound source in a mixture helps both to voluntarily attend and to ignore this source. Predictability is thus a *symmetric* cue, which contrasts Bregman's (1990, p. 669) definition of higher-level cues in ASA.

A symmetry test at a different level concerns the question as to whether predictability can support not only stream segregation but also integration. One recent study (Bendixen et al., in revision) provided the so far missing evidence that selectively enhancing predictability of the “Integrated” organization leads to an increase in perceptual reports of stream integration—the counterpart of what was demonstrated for stream segregation many times before. The data of Rajendran et al. (2013) can also be interpreted in this way, although segregated and integrated predictability were not strictly disentangled in this study. Hence predictability can now be regarded as a *symmetric* cue on multiple levels, in that it flexibly supports either stream segregation or integration, and in that it is effective both when it is present in an attended foreground sound source and when it is present in an unattended background sound source.

The latter finding is qualified by the results of Rimmele et al. (2012) who demonstrated that the facilitation of ASA by predictability in a background sound source shows age-related decline. These authors applied an objective-listening task requiring stream segregation. In a between-subject design, older listeners (mean age of 67 years) were shown to benefit less from background predictability than younger listeners (mean age of 22 years). In contrast, the effect of foreground predictability was similar in both age groups. The authors explain their finding by the fact that older listeners are known to exhibit deficits in predictability extraction from sequences outside the focus of attention (cf. reviews by Pekkonen, 2000; Näätänen et al., 2012). It should, however, be conceded that age-related impairments in ASA were observed for only one out of two types of background predictability, with the reason for this difference between predictability conditions remaining unclear. Moreover, Rimmele et al. (2012) did not record measures of predictability extraction in their participants. Hence although the interpretation that impairments in predictability extraction translate into corresponding impairments in predictability-based stream segregation is suggestive, this link remains to be directly examined. If the assumed relation between predictability extraction capacities and the ability to exploit predictability for ASA could be demonstrated at a single-subject level, this would have strong implications for models of impaired ASA (e.g., Beutelmann et al., 2010).

Whether there is or is not a direct link with predictability extraction, one can conclude from the data of Rimmele et al. (2012) that there are age-related difficulties in predictability-related aspects of ASA when predictability needs to be processed outside the focus of attention. This contrasts previous observations of sequential ASA being preserved in older participants (Trainor and Trehub, 1989; Snyder and Alain, 2007a), which have led to the assumption that only concurrent ASA declines with age (Snyder and Alain, 2005; Alain and McDonald, 2007). The data of Rimmele et al. (2012; see also Hutka et al., 2012) might thus help in filling the gap between the troublesome hearing impairments experienced by older listeners (e.g., Schneider et al., 2002; Shinn-Cunningham and Best, 2008) and the surprising failure to replicate such difficulties in the laboratory. If future studies confirm the notion that some aspects of sequential ASA do show an age-related decline, this insight has the potential to inform hearing aid design or to develop training programs for counteracting such deficits.

## Understanding predictability effects: perception or attention?

Given that effects of predictability on ASA have now been consistently demonstrated across studies and laboratories, a next important step is to understand *how* predictability affects ASA. One imminent question pertains to whether all the above-reviewed studies indeed measured predictability effects on the perception of the tone sequences (i.e., on the processes of stream segregation and integration), or whether other aspects of auditory processing might have been influenced as well. For the objective-listening tasks, it is possible that predictability facilitated processes of attention rather than sound organization. It has already been argued by Bregman (1990) that predictable sequences are easier to hold in the focus of attention (cf. Jones and Boltz, 1989). Somewhat more cumbersome but still possible, one may also posit that a predictable sequence in the background is easier to shield from attentive processing in order to spare resources for foreground listening (Devergie et al., 2010). How the brain uses predictability for directing attention toward or away from incoming stimuli is an area of active exploration (for a recent review, see Henry and Herrmann, 2014). When considering objective-listening tests alone, one could thus argue that predictability benefits in task performance stem from processes that are unrelated to sound organization.

However, when considering the objective-listening together with the subjective-report data, this interpretation becomes less likely: The same predictability manipulation was shown to increase subjective perceptual reports of “Segregation” (without specific instruction in terms of attention) and to improve performance in a task designed to require a “Segregated” percept. This makes the interpretation that the predictability manipulation exerted its influence through acting upon auditory perceptual organization much more plausible. As pointed out above, the two approaches complement each other in the type of inference they allow.

Further evidence for the view that predictability effects on stream segregation comprise more than attentional allocation comes from a recent MMN study (Bendixen et al., 2012b). This study showed that a series of interleaved tone sequences could be disentangled solely on the basis of predictability when the sequences were presented outside the focus of attention. In contrast, listeners mostly failed to segregate the streams when attentively trying to do so. This failure during active listening makes it highly unlikely that attention could have contributed to the predictability effect during passive listening. Hence the “pre-attentive” (Sussman, 2007) auditory system appears to be equipped with a bottom-up mechanism disentangling a mixture of two sound streams solely based on the predictability of these streams.

Taken together, these results suggest that predictability is effortlessly taken into account for auditory perceptual organization and does not require attentive processing of the predictable sound source, nor of the predictability itself. This conclusion is consistent with the view of predictive processing as the “default mode” of the brain (Friston, 2005, 2010), and it exceeds Bregman's (1990) framework in which the role of predictability was confined to an attention-mediated stage of ASA.

The consistent results from objective and subjective listening procedures nevertheless do not imply that predictability effects measured in objective-listening tests are *entirely* attributable to processes of sound organization rather than attention. It remains possible that both effects contribute (cf. Lange, 2013), and this possibility should be carefully considered for each objective-listening study. This seems particularly important for the potentially translational perspective of understanding predictability-related deficits in older listeners (Rimmele et al., 2012). Difficulties of older listeners in the allocation of auditory attention are well described (e.g., Mager et al., 2005; Horváth et al., 2009; Lawo and Koch, in press), and hence they must be examined as a viable alternative explanation.

## Understanding predictability effects: early or late?

When asking *how* predictability affects sound organization processes in ASA, another imminent question pertains to whether it acts as an early grouping cue (affecting the initial decomposition of the auditory input) or as a late grouping cue (confirming or disconfirming the decomposition provided by the early cues). As outlined above, the studies employing subjective-report procedures can shed some further light on this. Analyzing the switching characteristics between the perceptual reports of “Integration” and “Segregation” offers a straightforward way of determining whether a given cue induces or merely stabilizes the associated perceptual organization.

Initial results with this method (Bendixen et al., 2010) showed that a directional predictability cue stabilized the “Segregated” perceptual organization (i.e., it prolonged the duration of “Segregated” percepts), but it did not cause switching toward a “Segregated” perceptual organization (i.e., it did not shorten the duration of “Integrated” reports due to perception switching back to “Segregation”). Within a two-stage framework of auditory scene analysis (Bregman, 1990), these results suggest that predictability does not act upon the first stage during which the auditory input is decomposed, but only upon the second stage during which the sound groups are evaluated. The effect of predictability could be conceptualized as giving support to the configurations provided by the first stage depending on how successfully they predict newly incoming sounds. The decomposition itself would be dominated by primary acoustic features (such as spectral separation) exerting their influence in the first stage.

This interpretation received further support from a study with an orthogonal manipulation of predictability and spectral separation (Bendixen et al., 2013a). This study showed qualitatively different effects of manipulating these two types of cues, in that predictability again had stabilizing effects on “Segregated” perceptual reports, whereas spectral separation not only stabilized but also induced stream segregation. Additional support for a dissociation between the cues was provided by the absence of statistical interactions between the effects of spectral separation and predictability. Furthermore, the two types of cues responded differently to changes introduced during the sequence: Changing spectral separation was associated with a contrastive carry-over effect (similar to Snyder et al., 2009a,b), while changing predictability led to no carry-over effect. These findings were taken to suggest that predictability exerts its influence upon ASA only after primary acoustic grouping cues have been considered (Bendixen et al., 2010, 2013a,b). This implies that the impact of predictability is not as large as theoretically possible when deriving hypotheses from a strict predictive-processing point of view (Friston, 2005, 2010). In particular, there is no compelling support for the idea that predictability could be the earliest grouping cue (based on its availability with stimulus onset) or that it acts in parallel with the acoustic grouping cues.

This initially clear-cut inference must be viewed with more caution now that more evidence regarding predictability effects in ASA has been gathered. First, there are now demonstrations of interactions between spectral separation and predictability (Andreou et al., 2011). Second, there is ERP (though no behavioral) evidence that stream-specific predictable patterns can induce stream segregation when spectral separation and other primary feature differences between the streams are removed (Bendixen et al., 2012b). Third, switching characteristics for predictability manipulations in a more recent study (Bendixen et al., in revision) showed not only inducing but also stabilizing properties (see also Szalárdy et al., in press, for a predictability-like manipulation based on musical structure). Hence, although the question of *how* predictability acts upon ASA received a seemingly simple answer initially, it appears more appropriate to view this as an issue for further investigation.

A possible explanation for the discrepant results comes from another recent line of studies on the so-called *hierarchical novelty detection* system (cf. reviews by Grimm and Escera, 2012; Escera et al., in press). These authors show that violations of simple-repetition types of predictability are detected by the auditory system considerably earlier (in the middle-latency range) than violations of complex-pattern types of predictability. Complex predictability (such as a regular alternation between two feature values: “121212…”) does not affect processing until the MMN latency range (cf. Cornella et al., 2012; Althen et al., 2013). This implies that predictability information in terms of stimulus repetition is available to the auditory system as early as 20 ms post-stimulus while information in terms of more complex patterns is available only later in auditory processing, at around 150 ms post-stimulus. In other words, although the information about the next (predictable) sound is in theory available before stimulus onset once the predictability is extracted, the auditory system does not actually “pass down” this information from cortical structures to earlier processing stages. If this is the case, then it is not surprising to see that such complex forms of predictability cannot act upon ASA at early time-points. Together with the fact that stream formation based on strong acoustic cues (e.g., large spectral separation) occurs within less than 100 ms (Müller et al., 2005) and probably starts in peripheral structures of the auditory pathway (Pressnitzer et al., 2008), complex forms of predictability would be bound to exert their influence only after the acoustic cues have been taken into account.

In that respect, it is worthy to note that those studies showing that predictability acts upon ASA *after* the primary features (Bendixen et al., 2010, 2013a) employed complex-pattern types of predictability. In contrast, those studies whose results suggest that predictability acts at least in parallel with the primary features (Andreou et al., 2011; Bendixen et al., 2012b; in revision) have employed simple forms of predictability based on repetition or gradual progression of feature values. It is thus possible that simple types of predictability show early effects in ASA whereas complex forms of predictability are indeed limited to acting at a later stage. The interpretation that some forms of predictability affect auditory processing earlier than other forms is consistent with the view that stream formation can be triggered on various levels of the auditory processing hierarchy depending on the types of available cues (Cusack et al., 2004; Griffiths and Warren, 2004; Snyder and Alain, 2007b; Pressnitzer et al., 2008). Yet a direct comparison of the effects of predictability on different levels of complexity remains to be performed in future studies.

## Putting the role of predictability into perspective

Although the precise mechanisms are not yet specified, the studies reviewed above clearly demonstrate that predictability affects ASA. These findings lend support to theories linking predictive processing and ASA (Denham and Winkler, 2006; Winkler, 2007; Winkler et al., 2009), and they have encouraged the formulation of further conceptual and computational models of ASA based on predictive-processing principles (Winkler and Czigler, 2012; Winkler et al., 2012; Mill et al., 2013; Schröger et al., in press). Such efforts should not be mistaken to suggest that strict predictability is necessary for stream formation to occur. It has been shown several times that with unpredictable tone arrangements, stream formation simply operates on the basis of other cues (French-St. George and Bregman, 1989; Müller et al., 2005; Denham et al., 2010; Carl and Gutschalk, 2013). In other words, auditory perception readily tolerates random variation in the signal emissions of putative sound sources.

Sound organizations with predictable behavior of the resulting sound sources are sizably but not exclusively preferred over random arrangements. This is evidenced by the sometimes modest effect sizes of predictability manipulations (e.g., ~10% change in perceptual reports in Bendixen et al., 2010, 2013a). In order to put such numbers into perspective, it is important to acknowledge that the labels “predictable” and “unpredictable” exaggerate the differences between conditions in many studies. This is because tones in the “unpredictable” arrangement are usually still predictable in many properties. Typically, only one or two tone parameters are manipulated, and the lack of predictability is caused by random choice between a handful of feature values. Hence it may be more appropriate to see the above-reviewed studies as comparing predictability in a strict sense with predictability in a wider sense. With this in mind, it becomes more remarkable that such robust effects of predictability were achieved.

The predictability effects are consistent with the results of MMN studies showing that the auditory system prefers deterministic over stochastic sequential structures (Winkler et al., 1990; Schröger and Roeber, 2011; Daikhin and Ahissar, 2012). Knowing that one out of two possible feature values will come next in a sequence seems to be less beneficial than knowing exactly which one. In other words, ASA differentiates between the exact continuation and the inexact but plausible continuation of a stream. This provides another level of distinction between the role of predictability and earlier effects described for the Gestalt principles of *similarity* and *continuity* (Bregman, 1990). It also provides another case where predictability effects in ASA follow the same principles as auditory predictive processing *per se*, illustrating the prospects of combining these research lines (Denham and Winkler, 2006; Winkler et al., 2009, 2012; Schröger et al., in press).

## Predictability as a sequential analogue to the simultaneous old-plus-new heuristic?

Starting with the notion of a sequential analogue to the simultaneous *old-plus-new heuristic*, this review explored the idea that the auditory system's capacity to detect predictability in a sequence of discrete sounds could be used to support sequential ASA. Such supportive effects are clearly apparent in the reviewed studies. Sequential ASA shows a bias for perceptual interpretations comprising “old” (previously identified, well-describable and thus predictable) behavior of sound sources over interpretations including “new” signal emissions (i.e., unpredictable, surprising signals that are not yet characterized or not possible to characterize within the limitations of auditory predictability extraction). A further effect not explored in the present review but corroborating the gist of “old-plus-new” in sequential ASA is the auditory system's tendency to maintain a current perceptual interpretation despite parameter changes (Sussman and Steinschneider, 2006; Rahne and Sussman, 2009; Snyder et al., 2009b).

Comparing the properties of predictability-related sequential ASA with the old-plus-new heuristic in simultaneous ASA, similarities and differences become apparent. Unlike assumed by Bregman (1990), sequential grouping distinguishes plausible continuation of a source's signal emissions (as expressed in the *similarity* and *continuity* Gestalt principles) from precise (i.e., predictable) continuation. This is equivalent to the expectation of precise signal continuation observed for simultaneous grouping in the old-plus-new heuristic (Darwin, 1995). However, the preference for precise continuation in simultaneous grouping is so strong that the expected continuation of a sound source is metaphorically subtracted from a mixture before applying any other decomposition principles (Bregman, 1990). In other words, the old-plus-new heuristic is the first grouping principle in simultaneous ASA, while the same does not seem to hold for sequential predictability. Despite this difference, it is probably helpful for theorizing to retain the notion of an analogy between predictability-related sequential ASA and the previously expressed old-plus-new heuristic in simultaneous grouping.

Interestingly in this respect, McDermott et al. (2011) proposed an extension of the old-plus-new heuristic in simultaneous ASA in a similar vein as suggested for the sequential analogue in the present review. Specifically, these authors propose that simultaneous grouping is facilitated by the recurrence of sound sources even if every single occurrence is accompanied by concurrently overlapping signals (so-called *embedded repetition*). This is supported by empirical evidence that repetition-driven extraction of sound sources is indeed possible (McDermott et al., 2011). The data of McDermott et al. (2011) relieve the constraint that the simultaneous old-plus-new heuristic only applies to uninterrupted continuation (Bregman, 1990). Some earlier findings can also be interpreted in this vein (Darwin et al., 1995; Hukin and Darwin, 1995; Shinn-Cunningham et al., 2007; Lee and Shinn-Cunningham, 2008): These studies showed that a few presentations of one element of a complex sound (i.e., non-embedded repetition) prior to the presentation of the whole sound can lead to segregation of the element from the complex (i.e., a case where sequential grouping overcomes the temporal coherence principle; Shamma et al., 2011). This effect has been called *sequential capture*, and has been discussed in the framework of (competitive) interactions between simultaneous and sequential grouping cues. Interpreting the prior findings as demonstrations of an extended old-plus-new effect on simultaneous grouping offers an alternative perspective that can also integrate the present findings: Interrupted but repeated occurrence of a sound source's signal emissions might be equally beneficial for sequential and for simultaneous ASA.

Although this might provide a compelling perspective of a ubiquitous old-plus-new heuristic, two important differences between the respective sequential and simultaneous phenomena should not be neglected. First, old-plus-new effects on simultaneous ASA benefit from random changes in the concurrently present sources (McDermott et al., 2011) because in this case, the repetitive elements of the “old” source can be extracted more easily. In contrast, the corresponding effect in sequential ASA benefits from both sources being fully predictable (Bendixen et al., 2012b), lending itself more readily to a predictive-coding-based explanation.

Second, old-plus-new effects on simultaneous ASA have mainly been demonstrated for simple repetition (see also Best et al., 2008; Dyson and Alain, 2008), and have been shown to break down rapidly with more complex forms of predictability (Jones and Litovsky, 2008; Best et al., 2010). In contrast, sequential old-plus-new effects occur with predictable patterns of sound events exceeding mere repetition, as reviewed above. For postulating a common underlying principle, old-plus-new effects on the segregation of simultaneous sound sources would need to be demonstrated with regularities on different levels of complexity. Altogether, a further exploration into analogies of the old-plus-new heuristic in simultaneous and sequential sound grouping is expected to yield further insights into both types of grouping.

## Outstanding issues

Now that the general case for predictability effects in ASA appears to be made, it is time for a more detailed investigation into the involved mechanisms to gain a better understanding of their properties and limitations. Hence this review will end with a set of open questions and issues to be explored in future studies.

### Role of the type of predictability

As outlined above, future studies should explicitly contrast the effects of simple and complex regularities and investigate whether this affects the time-range at which predictability exerts its influence. This should be done while bearing in mind that for simple-repetition regularities, it is all the more difficult to separately manipulate predictability of the “Segregated” and “Integrated” organizations. Moreover, simple-repetition regularities typically confound predictability with the amount of feature variation, since the unpredictable condition used for comparison is unavoidably more variable in the feature values (i.e., the unpredictable tones are more dissimilar to each other than the predictable ones). Notwithstanding these interpretational difficulties, studying predictability effects on different levels of complexity is needed to settle the above-mentioned controversy regarding the timescale of the predictability effects on sequential ASA.

### Role of the features carrying the regularities

Predictability was manipulated by means of different stimulus features in the reviewed studies, including frequency, intensity, location, timing, and some combinations of these. Future studies should attempt to distinguish between the effects of these different features, as they are regarded as qualitatively distinct in theoretical frameworks of auditory processing. Näätänen and Picton (1987) coined the terms *temporal uncertainty* and *event uncertainty* to emphasize the qualitative difference between temporal regularities (reflecting the *when* aspect) and feature regularities (reflecting the *what* aspect; see also recent work by Sperduti et al., 2011; Arnal and Giraud, 2012; Hughes et al., 2013; Schwartze et al., 2013). Location, reflecting the *where* aspect, may constitute yet another qualitatively different feature (Rauschecker and Tian, 2000; Schwartz and Shinn-Cunningham, 2010). Thus, although the different features were initially treated as somewhat interchangeable to demonstrate the general principle of predictability effects in ASA, their specific effects should be disentangled in future studies (for an analogy in simultaneous ASA, see Kitterick et al., 2010).

### Mechanisms underlying predictability-related ASA processes

The mechanisms supporting predictability effects in ASA are far from being understood. Further studies will be required to clarity the controversies alluded to above (perceptual vs. attentional mechanisms as well as early vs. late effects on perceptual organization). It would be desirable for these studies to receive further inspiration from the field of predictive processing, including the ever-growing understanding of how predictability is instantiated in neuronal terms (e.g., Besle et al., 2011; Arnal and Giraud, 2012; Wacongne et al., 2012), in order to generate more precise hypotheses as to how predictability might act within ASA.

### Relation of predictability with other grouping cues

Obviously, predictability effects should not be treated in isolation but integrated into a comprehensive model of ASA. Integrating predictability into a general ASA framework will also require thorough investigation of how predictability effects relate to those of other grouping cues, both primary acoustic ones (e.g., spectral separation) and higher-level cues (e.g., familiarity with a sound source, cf. Johnsrude et al., 2013).

### Age effects

In view of the practical implications, it seems of utmost importance to understand the origin of the age-related decline in predictability-based ASA. A highly relevant question is how much this effect actually contributes to age-related deficits in ASA in challenging real-life listening situations. This immediately relates to the next and final outstanding issue.

### Transfer to natural listening conditions

It would be highly desirable to show a predictability benefit for ASA with more natural stimulus material; in other words, to demonstrate the ecological validity of the findings reviewed here. To what extent do listeners exploit natural predictability for forming a stable auditory stream of their conversation partner in a noisy environment? Natural auditory scenes obviously contain less strict forms of predictability, and the issue as to how much variability the brain tolerates while still treating a sound source's signal emissions as predictable is largely unresolved (but see Winkler et al., 1990; Daikhin and Ahissar, 2012). This issue must be addressed, however, if the present results shall be transferred to more applied domains. A perceptually inspired approach toward predictability in ASA might be highly informative for technical approaches of noise cancellation by predictive principles (e.g., Guldenschuh and Höldrich, 2013).

## Conclusions

In summary, there is a growing body of evidence from studies with complementary methods demonstrating that the impact of sound predictability on ASA is considerably more extensive than previously assumed. First, perceptual grouping favors precise continuation of a sound source's signal emissions over inexact but plausible (i.e., within the range of the sound source's feature characteristics) continuation; predictability thereby differs from the Gestalt principles of *similarity* and *continuity*. Second, effects of predictability on ASA need not be mediated by attention, but are readily explained in a bottom-up framework of automatically extracting predictability and applying it for further processing. Third, effects of predictability on ASA are symmetric: Maintaining segregation of a foreground and background stream is facilitated by predictability in either stream. Fourth, predictability acts as a symmetric cue also with respect to supporting either stream segregation or integration. These effects can be observed only if *directional* manipulations are employed: Predictability of the “Segregated” and “Integrated” perceptual organizations must be disentangled in the experimental design to allow for unambiguous interpretations. The precise mechanisms for predictability effects in ASA as well as for possible age-related impairments of these effects remain to be determined. Deriving a joint theoretical framework for sequential predictability and the simultaneous old-plus-new heuristic that receives further inspiration from the field of auditory predictive processing is considered a promising avenue for future research.

### Conflict of interest statement

The author declares that the research was conducted in the absence of any commercial or financial relationships that could be construed as a potential conflict of interest.

## References

[B1] Alain C. McDonald K. L. (2007). Age-related differences in neuromagnetic brain activity underlying concurrent sound perception. J. Neurosci. 27, 1308–1314 10.1523/JNEUROSCI.5433-06.2007 17287505 PMC6673581

[B2] Althen H. Grimm S. Escera C. (2013). Simple and complex acoustic regularities are encoded at different levels of the auditory hierarchy. Eur. J. Neurosci. 38, 3448–3455 10.1111/ejn.12346 23992232

[B3] Andreou L.-V. Kashino M. Chait M. (2011). The role of temporal regularity in auditory segregation. Hear. Res. 280, 228–235 10.1016/j.heares.2011.06.001 21683778

[B4] Anstis S. Saida S. (1985). Adaptation to auditory streaming of frequency-modulated tones. J. Exp. Psychol. Hum. Percept. Perform. 11, 257–271 10.1037/0096-1523.11.3.257

[B5] Arnal L. H. Giraud A.-L. (2012). Cortical oscillations and sensory predictions. Trends Cogn. Sci. 16, 390–398 10.1016/j.tics.2012.05.003 22682813

[B6] Baldeweg T. (2006). Repetition effects to sounds: evidence for predictive coding in the auditory system. Trends Cogn. Sci. 10, 93–94 10.1016/j.tics.2006.01.010 16460994

[B7] Bendixen A. Andersen S. K. (2013). Measuring target detection performance in paradigms with high event rates. Clin. Neurophysiol. 124, 928–940 10.1016/j.clinph.2012.11.012 23266090

[B8] Bendixen A. Bőhm T. M. Szalárdy O. Mill R. Denham S. L. Winkler I. (2013a). Different roles of similarity and predictability in auditory stream segregation. Learn. Percept. 5, 37–54 10.1556/LP.5.2013.Suppl2.4

[B9] Bendixen A. Denham S. L. Gyimesi K. Winkler I. (2010). Regular patterns stabilize auditory streams. J. Acoust. Soc. Am. 128, 3658–3666 10.1121/1.3500695 21218898

[B11] Bendixen A. Denham S. L. Winkler I. (2013b). Sound predictability as a higher-order cue in auditory scene analysis, in AIA-DAGA 2013, International Conference on Acoustics (Berlin: DEGA), 705–708

[B12] Bendixen A. SanMiguel I. Schröger E. (2012a). Early electrophysiological indicators for predictive processing in audition: a review. Int. J. Psychophysiol. 83, 120–131 10.1016/j.ijpsycho.2011.08.003 21867734

[B13] Bendixen A. Schröger E. Ritter W. Winkler I. (2012b). Regularity extraction from non-adjacent sounds. Front. Psychol. 3:143 10.3389/fpsyg.2012.00143 22661959 PMC3356878

[B14] Bendixen A. Schröger E. Winkler I. (2009). I heard that coming: event-related potential evidence for stimulus-driven prediction in the auditory system. J. Neurosci. 29, 8447–8451 10.1523/Jneurosci.1493-09.2009 19571135 PMC6665649

[B15] Besle J. Schevon C. A. Mehta A. D. Lakatos P. Goodman R. R. McKhann G. M. (2011). Tuning of the human neocortex to the temporal dynamics of attended events. J. Neurosci. 31, 3176–3185 10.1523/JNEUROSCI.4518-10.2011 21368029 PMC3081726

[B16] Best V. Ozmeral E. J. Kopčo N. Shinn-Cunningham B. G. (2008). Object continuity enhances selective auditory attention. Proc. Natl. Acad. Sci. U.S.A. 105, 13174–13178 10.1073/pnas.0803718105 18719099 PMC2529120

[B17] Best V. Shinn-Cunningham B. G. Ozmeral E. J. Kopčo N. (2010). Exploring the benefit of auditory spatial continuity. J. Acoust. Soc. Am. 127, EL258–EL264 10.1121/1.3431093 20550229 PMC2887909

[B18] Beutelmann R. Brand T. Kollmeier B. (2010). Revision, extension, and evaluation of a binaural speech intelligibility model. J. Acoust. Soc. Am. 127, 2479–2497 10.1121/1.3295575 20370031

[B19] Bey C. McAdams S. (2002). Schema-based processing in auditory scene analysis. Percept. Psychophys. 64, 844–854 10.3758/BF03194750 12201342

[B20] Boh B. Herholz S. C. Lappe C. Pantev C. (2011). Processing of complex auditory patterns in musicians and nonmusicians. PLoS ONE 6:e21458 10.1371/journal.pone.0021458 21750713 PMC3131276

[B21] Bregman A. S. (1990). Auditory Scene Analysis. The Perceptual Organization of Sound. Cambridge, MA: MIT Press

[B22] Carl D. Gutschalk A. (2013). Role of pattern, regularity, and silent intervals in auditory stream segregation based on inter-aural time differences. Exp. Brain Res. 224, 557–570 10.1007/s00221-012-3333-z 23161159

[B23] Cherry E. C. (1953). Some experiments on the recognition of speech, with one and with two ears. J. Acoust. Soc. Am. 25, 975–979 10.1121/1.1907229

[B24] Cornella M. Leung S. Grimm S. Escera C. (2012). Detection of simple and pattern regularity violations occurs at different levels of the auditory hierarchy. PLoS ONE 7:e43604 10.1371/journal.pone.0043604 22916282 PMC3423368

[B25] Coy A. Barker J. (2007). An automatic speech recognition system based on the scene analysis account of auditory perception. Speech Commun. 49, 384–401 10.1016/j.specom.2006.11.002

[B26] Cusack R. Deeks J. Aikman G. Carlyon R. P. (2004). Effects of location, frequency region, and time course of selective attention on auditory scene analysis. J. Exp. Psychol. Hum. Percept. Perform. 30, 643–656 10.1037/0096-1523.30.4.643 15301615

[B27] Daikhin L. Ahissar M. (2012). Responses to deviants are modulated by subthreshold variability of the standard. Psychophysiology 49, 31–42 10.1111/j.1469-8986.2011.01274.x 21899557 PMC3240736

[B28] Darwin C. J. (1995). Perceiving vowels in the presence of another sound: a quantitative test of the ‘old-plus-new’ heuristic, in Levels in Speech Communication: Relations and Interactions: A Tribute to Max Wajskop, eds Sorin C. Mariani J. Méloni H. Schoentgen J. (Amsterdam: Elsevier), 1–12

[B29] Darwin C. J. Hukin R. W. Al-Khatib B. Y. (1995). Grouping in pitch perception: Evidence for sequential constraints. J. Acoust. Soc. Am. 98, 880–885 10.1121/1.413513 7642826

[B30] Denham S. L. Bőhm T. M. Bendixen A. Szalárdy O. Kocsis Z. Mill R. (2014). Stable individual characteristics in the perception of multiple embedded patterns in multistable auditory stimuli. Front. Neurosci. 8:25 10.3389/fnins.2014.00025 24616656 PMC3937586

[B31] Denham S. L. Gyimesi K. Stefanics G. Winkler I. (2010). Stability of perceptual organisation in auditory streaming, in The Neurophysiological Bases of Auditory Perception, eds Lopez-Poveda E. A. Palmer A. R. Meddis R. (New York, NY: Springer), 477–488 10.1007/978-1-4419-5686-6_44

[B32] Denham S. L. Gyimesi K. Stefanics G. Winkler I. (2013). Perceptual bi-stability in auditory streaming: how much do stimulus features matter? Learn. Percept. 5, 73–100 10.1556/LP.5.2013.Suppl2.6

[B33] Denham S. L. Winkler I. (2006). The role of predictive models in the formation of auditory streams. J. Physiol. Paris 100, 154–170 10.1016/j.jphysparis.2006.09.012 17084600

[B34] Devergie A. Grimault N. Tillmann B. Berthommier F. (2010). Effect of rhythmic attention on the segregation of interleaved melodies. J. Acoust. Soc. Am. 128, EL1–EL7 10.1121/1.3436498 20649182

[B35] Dowling W. J. Lung K. M.-T. Herrbold S. (1987). Aiming attention in pitch and time in the perception of interleaved melodies. Percept. Psychophys. 41, 642–656 10.3758/BF03210496 3615158

[B36] Drake C. Jones M. R. Baruch C. (2000). The development of rhythmic attending in auditory sequences: attunement, referent period, focal attending. Cognition 77, 251–288 10.1016/S0010-0277(00)00106-2 11018511

[B37] Dyson B. J. Alain C. (2008). It all sounds the same to me: Sequential ERP and behavioral effects during pitch and harmonicity judgments. Cogn. Affect. Behav. Neurosci. 8, 329–343 10.3758/CABN.8.3.329 18814469

[B38] Ellis D. P. W. (1999). Using knowledge to organize sound: the prediction-driven approach to computational auditory scene analysis and its application to speech/nonspeech mixtures. Speech Commun. 27, 281–298 10.1016/S0167-6393(98)00083-1

[B39] Escera C. Leung S. Grimm S. (in press). Deviance detection based on regularity encoding along the auditory hierarchy: Electrophysiological evidence in humans. Brain Topogr. 10.1007/s10548-013-0328-4 24218032

[B40] Friston K. (2005). A theory of cortical responses. Philos. Trans. R. Soc. Lond. B Biol. Sci. 360, 815–836 10.1098/rstb.2005.1622 15937014 PMC1569488

[B41] Friston K. (2010). The free-energy principle: a unified brain theory? Nat. Rev. Neurosci. 11, 127–138 10.1038/nrn2787 20068583

[B42] French-St. George M. Bregman A. S. (1989). Role of predictability of sequence in auditory stream segregation. Percept. Psychophys. 46, 384–386 10.3758/BF03204992 2798032

[B43] Giard M.-H. Perrin F. Pernier J. Bouchet P. (1990). Brain generators implicated in the processing of auditory stimulus deviance: a topographic event-related potential study. Psychophysiology 27, 627–640 10.1111/j.1469-8986.1990.tb03184.x 2100348

[B44] Godsmark D. Brown G. J. (1999). A blackboard architecture for computational auditory scene analysis. Speech Commun. 27, 351–366 10.1016/S0167-6393(98)00082-X

[B45] Gregory R. L. (1980). Perceptions as hypotheses. Philos. Trans. R. Soc. Lond. B Biol. Sci. 290, 181–197 10.1098/rstb.1980.0090 6106237

[B46] Griffiths T. D. Warren J. D. (2004). What is an auditory object? Nat. Rev. Neurosci. 5, 887–892 10.1038/nrn1538 15496866

[B47] Grimm S. Escera C. (2012). Auditory deviance detection revisited: evidence for a hierarchical novelty system. Int. J. Psychophysiol. 85, 88–92 10.1016/j.ijpsycho.2011.05.012 21669238

[B48] Grimm S. Escera C. Slabu L. Costa-Faidella J. (2011). Electrophysiological evidence for the hierarchical organization of auditory change detection in the human brain. Psychophysiology 48, 377–384 10.1111/j.1469-8986.2010.01073.x 20636288

[B49] Grossberg S. Govindarajan K. K. Wyse L. L. Cohen M. A. (2004). ARTSTREAM: a neural network model of auditory scene analysis and source segregation. Neural Netw. 17, 511–536 10.1016/j.neunet.2003.10.002 15109681

[B50] Guldenschuh M. Höldrich R. (2013). Prediction filter design for active noise cancellation headphones. IET Signal Process. 7, 497–504 10.1049/iet-spr.2012.0161

[B51] Gutschalk A. Micheyl C. Melcher J. R. Rupp A. Scherg M. Oxenham A. J. (2005). Neuromagnetic correlates of streaming in human auditory cortex. J. Neurosci. 25, 5382–5388 10.1523/JNEUROSCI.0347-05.2005 15930387 PMC1237040

[B52] Haykin S. Chen Z. (2005). The cocktail party problem. Neural Comput. 17, 1875–1902 10.1162/0899766054322964 15992485

[B53] Helmholtz H. (1859). On the Sensations of Tone as a Physiological Basis for the Theory of Music. 2nd English Edn. (Trans. Ellis A. J. 1885) Whitefish, MT: Reprinted by Kessinger Publishing, 2005

[B54] Henry M. J. Herrmann B. (2014). Low-frequency neural oscillations support dynamic attending in temporal context. Timing Time Percept. 2, 62–86 10.1163/22134468-00002011

[B55] Hill K. T. Bishop C. W. Yadav D. Miller L. M. (2011). Pattern of BOLD signal in auditory cortex relates acoustic response to perceptual streaming. BMC Neurosci. 12:85 10.1186/1471-2202-12-85 21849065 PMC3173374

[B56] Horváth J. Czigler I. Birkás E. Winkler I. Gervai J. (2009). Age-related differences in distraction and reorientation in an auditory task. Neurobiol. Aging 30, 1157–1172 10.1016/j.neurobiolaging.2007.10.003 18023507

[B57] Hughes G. Desantis A. Waszak F. (2013). Mechanisms of intentional binding and sensory attenuation: the role of temporal prediction, temporal control, identity prediction, and motor prediction. Psychol. Bull. 139, 133–151 10.1037/a0028566 22612280

[B58] Hukin R. W. Darwin C. J. (1995). Effects of contralateral presentation and of interaural time differences in segregating a harmonic from a vowel. J. Acoust. Soc. Am. 98, 1380–1387 10.1121/1.414348

[B59] Hupé J.-M. Pressnitzer D. (2012). The initial phase of auditory and visual scene analysis. Philos. Trans. R. Soc. Lond. B Biol. Sci. 367, 942–953 10.1098/rstb.2011.0368 22371616 PMC3282313

[B60] Hutka S. A. Alain C. Binns M. A. Bidelman G. M. (2012). Age-related differences in the sequential organization of speech sounds. J. Acoust. Soc. Am. 133, 4177–4187 10.1121/1.4802745 23742369

[B61] Johnsrude I. S. Mackey A. Hakyemez H. Alexander E. Trang H. P. Carlyon R. P. (2013). Swinging at a cocktail party: voice familiarity aids speech perception in the presence of a competing voice. Psychol. Sci. 24, 1995–2004 10.1177/0956797613482467 23985575

[B62] Jones G. L. Litovsky R. Y. (2008). Role of masker predictability in the cocktail party problem. J. Acoust. Soc. Am. 124, 3818–3830 10.1121/1.2996336 19206808 PMC2676623

[B63] Jones M. R. (1976). Time, our lost dimension: toward a new theory of perception, attention, and memory. Psychol. Rev. 83, 323–355 10.1037/0033-295X.83.5.323 794904

[B64] Jones M. R. Boltz M. (1989). Dynamic attending and responses to time. Psychol. Rev. 96, 459–491 10.1037/0033-295X.96.3.459 2756068

[B65] Jones M. R. Boltz M. Kidd G. (1982). Controlled attending as a function of melodic and temporal context. Percept. Psychophys. 32, 211–218 10.3758/BF03206225 7177759

[B66] Jones M. R. Kidd G. Wetzel R. (1981). Evidence for rhythmic attention. J. Exp. Psychol. Hum. Percept. Perform. 7, 1059–1073 10.1037/0096-1523.7.5.1059 6457108

[B67] Kitterick P. T. Bailey P. J. Summerfield A. Q. (2010). Benefits of knowing who, where, and when in multi-talker listening. J. Acoust. Soc. Am. 127, 2498–2508 10.1121/1.3327507 20370032

[B68] Köhler W. (1947). Gestalt Psychology: An Introduction to New Concepts in Modern Psychology. New York, NY: Liveright Publishing Corporation

[B69] Kondo H. M. Kashino M. (2009). Involvement of the thalamocortical loop in the spontaneous switching of percepts in auditory streaming. J. Neurosci. 29, 12695–12701 10.1523/JNEUROSCI.1549-09.2009 19812344 PMC6665088

[B70] Kondo H. M. Kitagawa N. Kitamura M. S. Koizumi A. Nomura M. Kashino M. (2012). Separability and commonality of auditory and visual bistable perception. Cereb. Cortex 22, 1912–1922 10.1093/cercor/bhr266 21965442

[B71] Lange K. (2013). The ups and downs of temporal orienting: a review of auditory temporal orienting studies and a model associating the heterogeneous findings on the auditory N1 with opposite effects of attention and prediction. Front. Hum. Neurosci. 7:263 10.3389/fnhum.2013.00263 23781186 PMC3678089

[B72] Lawo V. Koch I. (in press). Examining age-related differences in auditory attention control using a task-switching procedure. J. Gerontol. B Psychol. Sci. Soc. Sci. 10.1093/geronb/gbs107 23197343

[B73] Lee A. K. C. Shinn-Cunningham B. G. (2008). Effects of frequency disparities on trading of an ambiguous tone between two competing auditory objects. J. Acoust. Soc. Am. 123, 4340–4351 10.1121/1.2908282 18537385 PMC9014251

[B74] Lipp R. Kitterick P. Summerfield Q. Bailey P. J. Paul-Jordanov I. (2010). Concurrent sound segregation based on inharmonicity and onset asynchrony. Neuropsychologia 48, 1417–1425 10.1016/j.neuropsychologia.2010.01.009 20079754

[B75] Mager R. Falkenstein M. Störmer R. Brand S. Müller-Spahn F. Bullinger A. H. (2005). Auditory distraction in young and middle-aged adults: a behavioural and event-related potential study. J. Neural Transm. 112, 1165–1176 10.1007/s00702-004-0258-0 15614427

[B76] Masuda-Katsuse I. Kawahara H. (1999). Dynamic sound stream formation based on continuity of spectral change. Speech Commun. 27, 235–259 10.1016/S0167-6393(98)00084-3

[B77] May P. J. C. Tiitinen H. (2010). Mismatch negativity (MMN), the deviance-elicited auditory deflection, explained. Psychophysiology 47, 66–122 10.1111/j.1469-8986.2009.00856.x 19686538

[B78] McDermott J. H. Wrobleski D. Oxenham A. J. (2011). Recovering sound sources from embedded repetition. Proc. Natl. Acad. Sci. U.S.A. 108, 1188–1193 10.1073/pnas.1004765108 21199948 PMC3024660

[B79] McDonald K. L. Alain C. (2005). Contribution of harmonicity and location to auditory object formation in free field: evidence from event-related brain potentials. J. Acoust. Soc. Am. 118, 1593–1604 10.1121/1.2000747 16240820

[B80] Micheyl C. Oxenham A. J. (2010a). Objective and subjective psychophysical measures of auditory stream integration and segregation. J. Assoc. Res. Otolaryngol. 11, 709–724 10.1007/s10162-010-0227-2 20658165 PMC2975891

[B81] Micheyl C. Oxenham A. J. (2010b). Pitch, harmonicity and concurrent sound segregation: psychoacoustical and neurophysiological findings. Hear. Res. 266, 36–51 10.1016/j.heares.2009.09.012 19788920 PMC2885481

[B82] Mill R. W. Bőhm T. M. Bendixen A. Winkler I. Denham S. L. (2013). Modelling the emergence and dynamics of perceptual organisation in auditory streaming. PLoS Comput. Biol. 9:e1002925 10.1371/journal.pcbi.1002925 23516340 PMC3597549

[B83] Moore B. C. J. Gockel H. (2002). Factors influencing sequential stream segregation. Acta Acust. United Acust. 88, 320–333

[B84] Moore B. C. J. Gockel H. E. (2012). Properties of auditory stream formation. Philos. Trans. R. Soc. Lond. B Biol. Sci. 367, 919–931 10.1098/rstb.2011.0355 22371614 PMC3282308

[B85] Müller D. Widmann A. Schröger E. (2005). Auditory streaming affects the processing of successive deviant and standard sounds. Psychophysiology 42, 668–676 10.1111/j.1469-8986.2005.00355.x 16364062

[B86] Näätänen R. (1992). Attention and Brain Function. Hillsdale, NJ: Erlbaum

[B87] Näätänen R. Astikainen P. Ruusuvirta T. Huotilainen M. (2010). Automatic auditory intelligence: an expression of the sensory-cognitive core of cognitive processes. Brain Res. Rev. 64, 123–136 10.1016/j.brainresrev.2010.03.001 20298716

[B88] Näätänen R. Gaillard A. W. K. Mäntysalo S. (1978). Early selective-attention effect on evoked potential reinterpreted. Acta Psychol. 42, 313–329 10.1016/0001-6918(78)90006-9 685709

[B89] Näätänen R. Jacobsen T. Winkler I. (2005). Memory-based or afferent processes in mismatch negativity (MMN): a review of the evidence. Psychophysiology 42, 25–32 10.1111/j.1469-8986.2005.00256.x 15720578

[B90] Näätänen R. Kujala T. Escera C. Baldeweg T. Kreegipuu K. Carlson S. (2012). The mismatch negativity (MMN) - a unique window to disturbed central auditory processing in ageing and different clinical conditions. Clin. Neurophysiol. 123, 424–458 10.1016/j.clinph.2011.09.020 22169062

[B91] Näätänen R. Picton T. (1987). The N1 wave of the human electric and magnetic response to sound: a review and an analysis of the component structure. Psychophysiology 24, 375–425 10.1111/j.1469-8986.1987.tb00311.x 3615753

[B92] Näätänen R. Tervaniemi M. Sussman E. Paavilainen P. Winkler I. (2001). ‘Primitive intelligence’ in the auditory cortex. Trends Neurosci. 24, 283–288 10.1016/S0166-2236(00)01790-2 11311381

[B93] Nager W. Teder-Sälejärvi W. Kunze S. Münte T. F. (2003). Preattentive evaluation of multiple perceptual streams in human audition. Neuroreport 14, 871–874 10.1097/00001756-200305060-00019 12858050

[B94] Nelken I. (2004). Processing of complex stimuli and natural scenes in the auditory cortex. Curr. Opin. Neurobiol. 14, 474–480 10.1016/j.conb.2004.06.005 15321068

[B95] Opitz B. Rinne T. Mecklinger A. von Cramon D. Y. Schröger E. (2002). Differential contribution of frontal and temporal cortices to auditory change detection: fMRI and ERP results. Neuroimage 15, 167–174 10.1006/nimg.2001.0970 11771985

[B96] Pekkonen E. (2000). Mismatch negativity in aging and in Alzheimer's and Parkinson's diseases. Audiol. Neuro-Otol. 5, 216–224 10.1159/000013883 10859416

[B97] Picton T. W. Alain C. Otten L. Ritter W. Achim A. (2000). Mismatch negativity: different water in the same river. Audiol. Neuro-Otol. 5, 111–139 10.1159/000013875 10859408

[B98] Pressnitzer D. Hupé J.-M. (2006). Temporal dynamics of auditory and visual bistability reveal common principles of perceptual organization. Curr. Biol. 16, 1351–1357 10.1016/j.cub.2006.05.054 16824924

[B99] Pressnitzer D. Sayles M. Micheyl C. Winter I. M. (2008). Perceptual organization of sound begins in the auditory periphery. Curr. Biol. 18, 1124–1128 10.1016/j.cub.2008.06.053 18656355 PMC2559912

[B100] Pressnitzer D. Suied C. Shamma S. A. (2011). Auditory scene analysis: the sweet music of ambiguity. Front. Hum. Neurosci. 5:158 10.3389/fnhum.2011.00158 22174701 PMC3237025

[B101] Prinz W. (2006). What re-enactment earns us. Cortex 42, 515–517 10.1016/S0010-9452(08)70389-7 16881261

[B102] Rahne T. Sussman E. (2009). Neural representations of auditory input accommodate to the context in a dynamically changing acoustic environment. Eur. J. Neurosci. 29, 205–211 10.1111/j.1460-9568.2008.06561.x 19087164 PMC2649007

[B103] Rajendran V. G. Harper N. S. Willmore B. D. Hartmann W. M. Schnupp J. W. H. (2013). Temporal predictability as a grouping cue in the perception of auditory streams. J. Acoust. Soc. Am. 134, EL98–E104 10.1121/1.4811161 23862914 PMC4491984

[B104] Rauschecker J. P. Tian B. (2000). Mechanisms and streams for processing of “what” and “where” in auditory cortex. Proc. Natl. Acad. Sci. U.S.A. 97, 11800–11806 10.1073/pnas.97.22.11800 11050212 PMC34352

[B105] Rimmele J. M. Schröger E. Bendixen A. (2012). Age-related changes in the use of regular patterns for auditory scene analysis. Hear. Res. 289, 98–107 10.1016/j.heares.2012.04.006 22543088

[B106] Rogers W. L. Bregman A. S. (1993). An experimental evaluation of three theories of auditory stream segregation. Percept. Psychophys. 53, 179–189 10.3758/BF03211728 8433916

[B107] Scherg M. Vajsar J. Picton T. W. (1989). A source analysis of the late human auditory evoked potentials. J. Cogn. Neurosci. 1, 336–355 10.1162/jocn.1989.1.4.336 23971985

[B108] Schneider B. A. Daneman M. Pichora-Fuller M. K. (2002). Listening in aging adults: from discourse comprehension to psychoacoustics. Can. J. Exp. Psychol. 56, 139–152 10.1037/h0087392 12271745

[B109] Schröger E. Bendixen A. Denham S. L. Mill R. Bőhm T. M. Winkler I. (in press). Predictive regularity representations in violation detection and auditory stream segregation: from conceptual to computational models. Brain Topogr. 10.1007/s10548-013-0334-6 24271978

[B110] Schröger E. Roeber U. (2011). A difference in the brain's ability to automatically encode serially deterministic and stochastic regularities. Front. Hum. Neurosci. Conf. Abstr. XI Int. Conf. Cogn. Neurosci. 10.3389/conf.fnhum.2011.207.00031

[B111] Schubotz R. I. (2007). Prediction of external events with our motor system: towards a new framework. Trends Cogn. Sci. 11, 211–218 10.1016/j.tics.2007.02.006 17383218

[B112] Schwartz A. H. Shinn-Cunningham B. G. (2010). Dissociation of perceptual judgments of “what” and “where” in an ambiguous auditory scene. J. Acoust. Soc. Am. 128, 3041–3051 10.1121/1.3495942 21110599 PMC3003726

[B113] Schwartze M. Farrugia N. Kotz S. A. (2013). Dissociation of formal and temporal predictability in early auditory evoked potentials. Neuropsychologia 51, 320–325 10.1016/j.neuropsychologia.2012.09.037 23022431

[B114] Shamma S. A. Elhilali M. Micheyl C. (2011). Temporal coherence and attention in auditory scene analysis. Trends Neurosci. 34, 114–123 10.1016/j.tins.2010.11.002 21196054 PMC3073558

[B115] Shinn-Cunningham B. G. Best V. (2008). Selective attention in normal and impaired hearing. Trends Amplific. 12, 283–299 10.1177/1084713808325306 18974202 PMC2700845

[B116] Shinn-Cunningham B. G. Lee A. K. C. Oxenham A. J. (2007). A sound element gets lost in perceptual competition. Proc. Natl. Acad. Sci. U.S.A. 104, 12223–12227 10.1073/pnas.0704641104 17615235 PMC1924568

[B117] Snyder E. Hillyard S. A. (1976). Long-latency evoked potentials to irrelevant, deviant stimuli. Behav. Biol. 16, 319–331 10.1016/S0091-6773(76)91447-4 1275853

[B118] Snyder J. S. Alain C. (2005). Age-related changes in neural activity associated with concurrent vowel segregation. Cogn. Brain Res. 24, 492–499 10.1016/j.cogbrainres.2005.03.002 16099361

[B119] Snyder J. S. Alain C. (2007a). Sequential auditory scene analysis is preserved in normal aging adults. Cereb. Cortex 17, 501–512 10.1093/cercor/bhj175 16581981

[B120] Snyder J. S. Alain C. (2007b). Toward a neurophysiological theory of auditory stream segregation. Psychol. Bull. 133, 780–799 10.1037/0033-2909.133.5.780 17723030

[B121] Snyder J. S. Carter O. L. Hannon E. E. Alain C. (2009a). Adaptation reveals multiple levels of representation in auditory stream segregation. J. Exp. Psychol. Hum. Percept. Perform. 35, 1232–1244 10.1037/a0012741 19653761 PMC2726626

[B122] Snyder J. S. Holder W. T. Weintraub D. M. Carter O. L. Alain C. (2009b). Effects of prior stimulus and prior perception on neural correlates of auditory stream segregation. Psychophysiology 46, 1208–1215 10.1111/j.1469-8986.2009.00870.x 19674396

[B123] Sperduti M. Tallon-Baudry C. Hugueville L. Pouthas V. (2011). Time is more than a sensory feature: attenting to duration triggers specific anticipatory activity. Cogn. Neurosci. 2, 11–18 10.1080/17588928.2010.513433 24168420

[B124] Spielmann M. I. Schröger E. Kotz S. A. Bendixen A. (in press). Attention effects on auditory scene analysis: insights from event-related brain potentials. Psychol. Res. 10.1007/s00426-014-0547-7 24553776

[B125] Spielmann M. I. Schröger E. Kotz S. A. Pechmann T. Bendixen A. (2013). Using a staircase procedure for the objective measurement of auditory stream integration and segregation thresholds. Front. Psychol. 4:534 10.3389/fpsyg.2013.00534 23970873 PMC3747440

[B126] Sussman E. Ritter W. Vaughan H. G. Jr. (1999). An investigation of the auditory streaming effect using event-related brain potentials. Psychophysiology 36, 22–34 10.1017/S0048577299971056 10098377

[B127] Sussman E. Čeponienė R. Shestakova A. Näätänen R. Winkler I. (2001). Auditory stream segregation processes operate similarly in school-aged children and adults. Hear. Res. 153, 108–114 10.1016/S0378-5955(00)00261-6 11223301

[B128] Sussman E. S. (2007). A new view on the MMN and attention debate: the role of context in processing auditory events. J. Psychophysiol. 21, 164–175 10.1027/0269-8803.21.34.164

[B129] Sussman E. S. Bregman A. S. Wang W. J. Khan F. J. (2005). Attentional modulation of electrophysiological activity in auditory cortex for unattended sounds within multistream auditory environments. Cogn. Affect. Behav. Neurosci. 5, 93–110 10.3758/CABN.5.1.93 15913011

[B130] Sussman E. S. Gumenyuk V. (2005). Organization of sequential sounds in auditory memory. Neuroreport 16, 1519–1523 10.1097/01.wnr.0000177002.35193.4c 16110282

[B131] Sussman E. S. Horváth J. Winkler I. Orr M. (2007). The role of attention in the formation of auditory streams. Percept. Psychophys. 69, 136–152 10.3758/BF03194460 17515223

[B132] Sussman E. Steinschneider M. (2006). Neurophysiological evidence for context-dependent encoding of sensory input in human auditory cortex. Brain Res. 1075, 165–174 10.1016/j.brainres.2005.12.074 16460703 PMC2846765

[B133] Szalárdy O. Bendixen A. Bőhm T. M. Davies L. A. Denham S. L. Winkler I. (in press). The effects of rhythm and melody on auditory stream segregation. J. Acoust. Soc. Am. 10.1121/1.4865196 24606277

[B134] Szalárdy O. Bendixen A. Tóth D. Denham S. L. Winkler I. (2013). Modulation frequency acts as a primary cue for auditory stream segregation. Learn. Percept. 5, 149–161 10.1556/LP.5.2013.Suppl2.9

[B135] Tiitinen H. May P. Reinikainen K. Näätänen R. (1994). Attentive novelty detection in humans is governed by pre-attentive sensory memory. Nature 372, 90–92 10.1038/372090a0 7969425

[B136] Trainor L. J. Trehub S. E. (1989). Aging and auditory temporal sequencing: ordering the elements of repeating tone patterns. Percept. Psychophys. 45, 417–426 10.3758/BF03210715 2726404

[B137] Van Noorden L. P. A. S. (1975). Temporal Coherence in the Perception of Tone Sequences. Doctoral dissertation, Technical University Eindhoven, Eindhoven

[B138] Wacongne C. Changeux J. P. Dehaene S. (2012). A neuronal model of predictive coding accounting for the mismatch negativity. J. Neurosci. 32, 3665–3678 10.1523/JNEUROSCI.5003-11.2012 22423089 PMC6703454

[B139] Wertheimer M. (1923). Untersuchungen zur Lehre von der Gestalt II [Laws of organization in perceptual forms II]. Psychol. Forsch. 4, 301–350 10.1007/BF00410640

[B140] Winkler I. (2007). Interpreting the mismatch negativity. J. Psychophysiol. 21, 147–163 10.1027/0269-8803.21.34.147

[B141] Winkler I. Czigler I. (2012). Evidence from auditory and visual event-related potential (ERP) studies of deviance detection (MMN and vMMN) linking predictive coding theories and perceptual object representations. Int. J. Psychophysiol. 83, 132–143 10.1016/j.ijpsycho.2011.10.001 22047947

[B142] Winkler I. Denham S. L. Mill R. Bőhm T. M. Bendixen A. (2012). Multistability in auditory stream segregation: a predictive coding view. Philos. Trans. R. Soc. Lond. B Biol. Sci. 367, 1001–1012 10.1098/rstb.2011.0359 22371621 PMC3282310

[B143] Winkler I. Denham S. L. Nelken I. (2009). Modeling the auditory scene: predictive regularity representations and perceptual objects. Trends Cogn. Sci. 13, 532–540 10.1016/j.tics.2009.09.003 19828357

[B144] Winkler I. Karmos G. Näätänen R. (1996). Adaptive modeling of the unattended acoustic environment reflected in the mismatch negativity event-related potential. Brain Res. 742, 239–252 10.1016/S0006-8993(96)01008-6 9117400

[B145] Winkler I. Kushnerenko E. Horváth J. Čeponienė R. Fellman V. Huotilainen M. (2003a). Newborn infants can organize the auditory world. Proc. Natl. Acad. Sci. U.S.A. 100, 11812–11815 10.1073/pnas.2031891100 14500903 PMC208846

[B146] Winkler I. Paavilainen P. Alho K. Reinikainen K. Sams M. Näätänen R. (1990). The effect of small variation of the frequent auditory stimulus on the event-related brain potential to the infrequent stimulus. Psychophysiology 27, 228–235 10.1111/j.1469-8986.1990.tb00374.x 2247552

[B147] Winkler I. Sussman E. Tervaniemi M. Horváth J. Ritter W. Näätänen R. (2003b). Preattentive auditory context effects. Cogn. Affect. Behav. Neurosci. 3, 57–77 10.3758/CABN.3.1.57 12822599

[B148] Winkler I. Takegata R. Sussman E. (2005). Event-related brain potentials reveal multiple stages in the perceptual organization of sound. Cogn. Brain Res. 25, 291–299 10.1016/j.cogbrainres.2005.06.005 16005616

[B149] Winkler I. Teder-Sälejärvi W. A. Horváth J. Näätänen R. Sussman E. (2003c). Human auditory cortex tracks task-irrelevant sound sources. Neuroreport 14, 2053–2056 10.1097/00001756-200311140-00009 14600496

